# Anhedonia modulates benzodiazepine and opioid demand among persons in treatment for opioid use disorder

**DOI:** 10.3389/fpsyt.2023.1103739

**Published:** 2023-01-19

**Authors:** Mark K. Greenwald, Tabitha E. H. Moses, Leslie H. Lundahl, Timothy A. Roehrs

**Affiliations:** ^1^Substance Abuse Research Division, Department of Psychiatry and Behavioral Neurosciences, School of Medicine, Wayne State University, Detroit, MI, United States; ^2^Sleep Disorders Center, Henry Ford Health System, Detroit, MI, United States

**Keywords:** benzodiazepine, demand, anhedonia, affective dysregulation, opioid use disorder

## Abstract

**Background:**

Benzodiazepine (BZD) misuse is a significant public health problem, particularly in conjunction with opioid use, due to increased risks of overdose and death. One putative mechanism underlying BZD misuse is affective dysregulation, *via* exaggerated negative affect (e.g., anxiety, depression, stress-reactivity) and/or impaired positive affect (anhedonia). Similar to other misused substances, BZD consumption is sensitive to price and individual differences. Although purchase tasks and demand curve analysis can shed light on determinants of substance use, few studies have examined BZD demand, nor factors related to demand.

**Methods:**

This ongoing study is examining simulated economic demand for alprazolam (among BZD lifetime misusers based on self-report and DSM-5 diagnosis; *n* = 23 total; 14 male, 9 female) and each participant’s preferred-opioid/route using hypothetical purchase tasks among patients with opioid use disorder (*n* = 59 total; 38 male, 21 female) who are not clinically stable, i.e., defined as being early in treatment or in treatment longer but with recent substance use. Aims are to determine whether: (1) BZD misusers differ from never-misusers on preferred-opioid economic demand, affective dysregulation (using questionnaire and performance measures), insomnia/behavioral alertness, psychiatric diagnoses or medications, or urinalysis results; and (2) alprazolam demand among BZD misusers is related to affective dysregulation or other measures.

**Results:**

Lifetime BZD misuse is significantly (*p* < 0.05) related to current major depressive disorder diagnosis, opioid-negative and methadone-negative urinalysis, higher trait anxiety, greater self-reported affective dysregulation, and younger age, but not preferred-opioid demand or insomnia/behavioral alertness. Alprazolam and opioid demand are each significantly positively related to higher anhedonia and, to a lesser extent, depression symptoms but no other measures of negative-affective dysregulation, psychiatric conditions or medications (including opioid agonist therapy or inpatient/outpatient treatment modality), or sleep-related problems.

**Conclusion:**

Anhedonia (positive-affective deficit) robustly predicted increased BZD and opioid demand; these factors could modulate treatment response. Routine assessment and effective treatment of anhedonia in populations with concurrent opioid and sedative use disorder may improve treatment outcomes.

**Clinical trial registration:**

https://clinicaltrials.gov/ct2/show/NCT03696017, identifier NCT03696017.

## 1. Introduction

Although the opioid overdose epidemic continues to generate unprecedented numbers of deaths, medical, and epidemiological data clearly indicate these adverse outcomes are not solely due to over-consumption of opioids but often involve use of multiple substances (1–6). The Food and Drug Administration recognizes the health dangers of opioid/benzodiazepine (BZD) polysubstance use, and issued labeling changes for prescribing BZDs and opioids (7). However, the impact of such changes is minimal when people take a prescribed drug inconsistent with its labeling or use someone else’s prescription [e.g., (8)].

There has been limited systematic research on mechanisms underlying BZD/opioid polysubstance misuse [for review, (9)]. Although BZDs are often co-prescribed with opioids (10–13), there is substantial co-occurring use and misuse of opioids and BZDs (14–16). Whereas BZD misuse alone can be harmful, when combined with opioids, BZD misuse contributes dose-dependently to health-risk behaviors, poor treatment outcomes, overdoses and deaths (16–26).

Interpreting BZD misuse and consequences, particularly in the context of opioid misuse, is challenging. First, temporal patterns of opioid/BZD consumption are highly variable, ranging from simultaneous use (co-administration) to sequential use (one drug used within several hours before the other) to concurrent use (both drugs consumed during a broader temporal window, e.g., within a few days/weeks of one another). The behavioral mechanisms underlying these different co-use patterns are likely to differ. In fact, persons who co-use BZDs with opioids report several motives including managing anxiety, enhancing the drug “high,” promoting sleep, and suppressing opioid withdrawal (27–30). Second, BZD/opioid polysubstance use rarely occurs in isolation, i.e., persons using BZDs and opioids often use other psychoactive substances such as nicotine, alcohol, cannabis, and psychostimulants. Also, it is important to separate the psychopharmacological effects and consequences of BZD use from those of alcohol use, as these are highly comorbid (5, 31–33) and share similar reinforcing properties (34) and neurochemical mechanisms of action. A third interpretive challenge is that there are demographic differences in BZD/opioid polysubstance use. For example, BZD use and misuse is more common among women than men (26, 35), whites than other racial/ethnic groups (36), and among injection opioid users (26, 37); notably, the latter two factors are correlated, as some prior research has found opioid injectors are more likely to be white than black (38–40). Finally, several types of comorbidities can potentially modulate BZD/opioid use including anxiety-related symptoms/diagnoses (41–43), and sleep problems (9, 44, 45). A common assumption is that exaggerated negative affect plays a pivotal role in motivating BZD use to “self-medicate” anxious or depressive symptoms [i.e., negative reinforcement; (46)], however, this may not be the only functional relationship between psychiatric conditions and the reinforcing effects of BZDs.

Several theories of substance use disorders have outlined a central role of affective dysregulation and stress-reactivity (47–51). The present research builds on a dual-deficit theory of reward deficiency and stress surfeit in addiction (52). Our working hypothesis is that BZD/opioid polysubstance misuse may be perpetuated by a dual-deficit in hedonic regulation (difficulties modulating emotional reactions relative to the context and the person’s long-term goals). From the standpoint of clinical practice (which we emphasize more than etiological issues), we propose that this dual-deficit *maintains* polysubstance misuse and makes treatment more challenging. Further, we propose this dual-deficit biases motivated behaviors (predominantly guided by negative reinforcement processes), such that polysubstance use acutely blunts aversive states and directs actions away from natural rewards.

Benzodiazepine seeking/consumption, as for other misused substances, is sensitive to economic price. This process can be studied using self-administration (actual consumption) or hypothetical purchase tasks (simulated) and applying demand curve analysis to examine the intensity and elasticity of demand (53, 54), which can also be conceptualized as amplitude and persistence of demand, respectively (55). Alprazolam is a rapid-onset BZD that is frequently misused (56–59). Studies of rhesus monkeys have demonstrated that BZDs are self-administered, however, economic demand for BZDs is complexly related to a compound’s selectivity and intrinsic efficacy at α1 subunit-containing GABA_*A*_ receptors, as well as the animal’s baseline history of self-administration (60–64). Therefore, it is reasonable to use a standard, often-misused BZD such as alprazolam to investigate individual difference in BZD demand. Recently, it was shown that alprazolam functioned as a reinforcer in three of six monkeys tested. For two of those three alprazolam self-administering animals, alprazolam enhanced self-administration of fentanyl whereas for the other monkey alprazolam self-administration suppressed fentanyl intake (65). These data highlight the importance of individual differences in the reinforcing effects of BZDs and opioids; however, we presently have limited understanding of the reasons underlying these differences.

To our knowledge, only three clinical studies have used hypothetical purchasing tasks to investigate BZD demand, although none specifically with alprazolam. Petry and Bickel (66) studied 40 persons undergoing treatment for heroin use disorder. Among several price and income manipulations, they found that diazepam (the only BZD studied) substituted for heroin, whereas heroin purchases were independent of diazepam prices, suggesting an asymmetrical substitution effect. This indicates that diazepam is reinforcing in persons addicted to heroin but does not specify for what reason(s). In a separate study, Petry (67) also reported that diazepam demand was price-elastic among individuals with DSM-IV alcohol abuse/dependence and a history of polysubstance use. Recently, Schwartz et al. (68) studied 52 persons in outpatient opioid agonist treatment for opioid use disorder at a baseline visit and a 6-month follow-up visit; they found that demand intensity for BZD pills (not specified) increased across time points and was predictive of BZD-positive urine samples.

In summary, we lack data on factors that influence BZD demand, alone and especially in the context of opioid use disorder. Importantly, group factors can be included in demand curve analyses to examine individual difference variables that modulate BZD consumption. Accordingly, the present study aims to investigate: (1) among persons in treatment for opioid use disorder, whether lifetime or past-year BZD misusers differ from never-misusers on measures of simulated opioid demand (co-primary outcome), affective dysregulation (e.g., psychiatric diagnoses, anhedonia, distress tolerance), and insomnia/daytime sleepiness; and (2) in the subgroups of lifetime and past-year BZD misusers, whether simulated BZD demand (co-primary outcome) is specifically associated with affective dysregulation, controlling for other factors.

## 2. Materials and methods

### 2.1. Study context

The local IRB approved all research procedures. This ongoing study is being conducted according to the Declaration of Helsinki and is registered at ClinicalTrials.gov (NCT03696017). All participants provided informed consent.

### 2.2. Participant selection

This study assesses patients currently in treatment (baseline visit) for their opioid and potentially other substance use disorder(s) who are not presently clinically stable, which we defined *a priori* as early (first 6 months) in treatment or in treatment longer but self-report having used opioids during the past month. As this programmatic research is thematically focused on BZD/opioid polysubstance use, we attempted to recruit a sample enriched with individuals with a history of BZD misuse in addition to their opioid misuse; however, we did not explicitly require a history of, or current, use of BZDs to be enrolled in this study.

First, we defined *BZD misuse history* based on two lifetime factors, either: (1) any BZD misuse based on a “yes” response to the question, “Have you ever used sedatives/hypnotics not as prescribed intending to get high,” on the Drug History and Use Questionnaire DHUQ (described in Section “2.3.4. Substance use”), or (2) diagnosis of sedative use disorder involving a BZD based on the SCID diagnostic interview (described in Section “2.3.5. Psychopathology and affective dysregulation”). Any participant meeting at least one of these two criteria was classified as a lifetime BZD misuser, and any participant not meeting either criterion was classified as a BZD never-misuser. Importantly, any participant who reported using BZDs as exactly prescribed for them throughout their lifetime, and denied misuse, was classified as a never-misuser. Second, to account for possible temporal variation in the effects of BZD misuse or abstinence, we defined differences in *recency* of BZD misuse as either (1) more than 1 year ago, or (2) within the past year, relative to the date of the initial screening visit. Participants who reported BZD misuse more than 1 year prior, or met DSM-5 criteria for partially remitted or past sedative use disorder were classified as misusers more than a year ago. Participants who reported BZD misuse within the past year, or met DSM-5 criteria for current (past-year) sedative use disorder involving a BZD, were classified as past-year misusers. Thus, we formed three distinct groups for analyses: (1) never misuse, (2) misuse > 1 year ago, and (3) past-year misuse of BZDs.

All participants are adults, ages 18–70 years old enrolled in a substance use disorder treatment program (outpatient or residential) in the Detroit metropolitan region. Exclusion criteria were estimated IQ < 80, expired breath alcohol > 0.02% breath alcohol concentration, neurological disorders that affect cognition, and current psychosis or suicidality. This study is also approved to re-contact participants (in-person or remotely) for 3-month follow-up assessment; these follow-up data will be reported elsewhere.

### 2.3. Experimental assessments

#### 2.3.1. Hypothetical opioid and benzodiazepine purchase tasks

A simulated *Opioid Purchasing Task* is tailored to each participant’s preferred opioid and route of administration (e.g., injected, snorted, oral) based on screening self-report. Of the 59 total participants, 46 reported using heroin (22 snorted, 23 injected, 1 smoked), 1 snorted fentanyl, 10 took oral hydrocodone, and 2 took oral oxycodone. The purchasing task is modeled after extant purchasing tasks for various substances [e.g., (69–71)], but personalizing the task for specific opioids/routes is novel. Participants are asked to imagine a typical day, with no access to other opioids unless they buy the preferred opioid at the listed prices. Participants make purchasing choices based on instructions that the amount purchased at each unit price (independent observations) must be consumed within 24-h (i.e., no saving or stockpiling drug). Prices per morphine 10-mg equivalent dose are $0 (free; no constraint) and 20 non-zero unit prices of $0.01, $0.10, $0.50, $1, $3, $5, $7.50, $10, $12.50, $15, $20, $25, $30, $35, $40, $45, $50, $60, $80, and $100. The participant indicates on a standard form how many unit doses s/he would purchase (dependent variable) at each unit price (independent variable).

A parallel simulated *BZD Purchasing Task* uses similar instructions and unit prices for alprazolam (0.25-mg equivalent oral dose): $0 (free), and $0.01, $0.10, $0.50, $1, $3, $5, $7.50, $10, $12.50, $15, $20, $25, $30, $35, $40, $45, $50, $60, $80, and $100. The participant indicates on a standard form how many unit doses s/he would purchase at each unit price. Among the 37 BZD misusers in this sample (11 of whom endorsed a prior prescription), 15 reported misuse of two or more BZDs across their lifetime (concurrent past-month misuse of multiple BZDs was infrequent): 25 endorsed ever misusing alprazolam (Xanax™), 13 endorsed misusing diazepam (Valium™), 11 endorsed misusing clonazepam (Klonipin™), and 6 endorsed misusing lorazepam (Ativan™), and 3 (who misused in the past month) did not identify the specific BZD(s) by name. All participants reported misuse of these BZDs only *via* the oral route of administration (e.g., no snorting or injection). Thus, use of an oral alprazolam purchasing task was appropriate in this participant sample.

#### 2.3.2. Demographics

Information on age, educational level/degree, and self-identified sex, race, and ethnicity are obtained *via* self-report. Estimated verbal intelligence is obtained by administering the Shipley Institute of Living Scale (72).

#### 2.3.3. Type of treatment

Standardized forms are used to collect information on type of treatment facility (acute or longer-term residential, transitional, day program, or other outpatient), type of medication for opioid use disorder [grouped as agonist therapy (methadone and buprenorphine) vs. no agonist therapy (naltrexone and no medication)], and other non-substance use disorder treatment medications (e.g., for anxiety, depression, sleep, pain).

#### 2.3.4. Substance use

Substance use is evaluated with a comprehensive *Drug History and Use Questionnaire* developed in our laboratory (available on request); it is used (either *via* paper/pencil or Qualtrics administration) to assess lifetime substance use (e.g., onset of use, regular use of opioids and BZDs and other substances, adverse consequences of substance use, number of quit attempts). This instrument also is used to determine the relative timeline of opioid and BZD use (prescribed or not), misuse and progression.

*Biomarkers of recent substance use* include alcohol breath testing and urine drug screening. Participants must provide a supervised alcohol-free breath sample (<0.02% BAC; AlcoSensor Intoximeter). A urine sample is collected into multi-test cups with temperature strips (CLIA Waived; temperature must be 92–96° F). Samples are tested for opioids, methadone, cocaine metabolites, benzodiazepines, amphetamines, barbiturates (negative cutoff < 300 ng/ml), and THC (negative cutoff < 50 ng/ml). After the study began, we initiated fentanyl urinalysis using test strips; however, at this time, too few participants have data for this measure.

#### 2.3.5. Psychopathology and affective dysregulation

The *Semi-Structured Clinical Interview for DSM-5* [SCID; (73, 74)] is used to evaluate lifetime and current psychiatric and substance use disorders. The SCID is administered by a trained clinical psychology masters level student, supervised by co-author LHL.

Anhedonia, the reduced experience or anticipation of pleasure (75, 76) linked to dopamine-mediated reward dysfunction and drug craving (77–80), is measured with the validated 14-item *Snaith-Hamilton Pleasure Scale* [*SHAPS*; (81, 82)]. Individuals are asked about their agreement with 14 statements; example items include: “I would be able to enjoy my favorite meal” (food/drink), “I would enjoy seeing others’ smiling faces” (social interaction), “I would be able to enjoy a beautiful landscape or view” (sensory experience), and “I would find pleasure in my hobbies and past-times” (interest/past-times), which is consistent with a recent conceptualization of anhedonia as having multiple domains, although these are not yet well understood (83). Each statement receives a score of either 0 (definitely agree or agree) or 1 (definitely disagree or disagree). High scores reflect the participant’s disagreement with the item statement (i.e., inability to experience pleasure from the event). Notably, most healthy individuals score < 2 (low anhedonia), whereas psychiatric patient samples often score 2 or higher.

The *Beck Depression Inventory-II (BDI-II*) is a gold-standard, 21-item clinical measure of current (past 2-week) depression symptoms validated against the original version (84) and in low-income African-Americans (85) and substance users (86). Guidelines for BDI-II cutoff scores are that: 0–13 indicates no or minimal depression; 14–19 indicates mild to moderate depression; 19–28 indicates moderate to severe depression; and 29–63 indicates severe depression (87).

The *State-Trait Anxiety Inventory (STAI)* (88) is a well-validated 40-item measure that differentiates symptoms of state anxiety (Y1 scale) from chronic trait anxiety (Y2 scale) by evaluating agreement with each item on a four-point Likert scale.

The 14-item *Perceived Stress Scale (PSS)* (89) measures the degree to which the subject views past-month life situations as stressful. It is reliable and correlates with self-report and behavioral criteria.

The 36-item *Difficulties with Emotion Regulation Scale (DERS)* (90) measures six empirically valid constructs related to emotion dysregulation: Non-acceptance of emotional responses, Difficulties in engaging in goal-directed behavior, Impulse control difficulties, Lack of emotional awareness, Limited access to emotion regulation strategies, and Lack of emotional clarity.

The 20-item *Alcohol and Drug Use Self-Efficacy Scale (ADUSE)* (91) assesses self-efficacy and responses to high-risk situations that can trigger substance use. Items are grouped into negative affect, social positive withdrawal/urges, and physical/other concerns; subjects indicate how “tempted” and “confident” they would be in each situation.

Distress tolerance, defined as the perceived capacity to tolerate distress and interpreted here as the ability to remain drug abstinent in the face of difficulties (92–94) is measured with the *Distress Tolerance Scale* which has 15 items with good construct validity and reliability (95).

We include two performance measures putatively related to affective dysregulation. The *Paced Auditory Serial Addition Task (PASAT)* (96) is a mental arithmetic task that measures processing speed and flexibility during which participants must add each new digit to the one presented immediately prior. We used three trial blocks of increasing difficulty such that the presentation rate of numbers that must be held in memory and added increases within each trial block. Participants can quit performing the task during trial block three; performance accuracy and latency to task termination are outcome measures. In the *Emotional Stroop Test* (97), words presented (in different colors) vary in their affective meaning: neutral, pleasant, negative, aggressive. The participant is instructed to identify (by key-pressing) the color of the printed word; response accuracy and latency (ms) are outcome measures.

#### 2.3.6. Insomnia and behavioral alertness

The 7-item *Insomnia Severity Index (ISI)* asks about problem severity of sleep-onset, sleep-maintenance, early morning awakening, sleep satisfaction, interference with daily function, perceived impairment, and level of distress from insomnia. It has good internal consistency and concurrent validity (with polysomnography, sleep diaries, and clinician or significant-other reports), making it a valid and reliable measure of perceived sleep disturbance (98, 99).

The 8-item *Epworth Sleepiness Scale (ESS)* measures “sleep propensity,” i.e., recent likelihood of dozing or falling asleep (rather than just feeling tired) in several situations (100). It is reliable and some items correlate with the gold-standard Multiple Sleep Latency Test.

The *Psychomotor Vigilance Task (PVT)* (101, 102) is a computerized, adaptive task (reaction time to a visual stimulus presented at random inter-trial intervals) that is used to assess attentional lapses; this objective, validated measure of sleepiness will complement the ISI and ESS measures.

### 2.4. Data analysis

Economic demand curve analysis is used to estimate the amounts of each participant’s preferred-opioid and, for lifetime BZD misusers, alprazolam consumed across increasing unit prices. Specifically, we measure each participant’s demand *intensity* (amplitude at low prices) and *elasticity* (resistance to price increases) based on the number of opioid $10 units purchased/consumed, in relation to opioid unit prices ranging from $0.01–$100.00. For participants with lifetime BZD misuse history, we also measure demand intensity and elasticity for alprazolam 0.25 mg units in relation to alprazolam unit prices, also ranging from $0.01–$100.00.

Hypothetical purchase task data were screened for unsystematic responses. Two curves that were unsystematic (one opioid, one BZD) were removed from analyses; this low proportion of data removal is similar to rates reported in prior studies. Each participant’s hypothetical purchase data were entered into a GraphPad Prism template^1^. Consumption values were transformed using the inverse hyperbolic sine transform (IHS; Equation 1 below) which is approximately log-equivalent for consumption values > 5 and for values < 5 converges to zero, such that zero consumption values can be included in analyses. Curves were fit with both non-normalized and normalized versions of the zero-bounded exponential model of demand (103):


IHS(Q)=IHS(Q)0*(e-[α÷IHS(Q)0]Qx0)

whereIHS(Q)0=log(0.5Q+00.25Q+021.10

In this model, *Q* is consumption, *Q*_0_ is consumption at unit price = 0 (demand *intensity*), *x* is unit price, and α is a free parameter that indexes the rate of change of the curve slope. This model accounts for these data which included many instances of reported zero consumption, and preserves the log-like scaling that represents relative changes in consumption with relative changes in unit price, i.e., the definition of *elasticity*.

Each model (opioid and BZD) was first used to estimate intensity of demand (*Q*_0_) and demand elasticity (α) and curve fit (*r*^2^), separately for each participant, in GraphPad Prism. The model also automatically calculates “essential value” [*EV*; (54)], which is proportional to the inverse of α [*EV* = 1/(100 × α)], and easily communicates the rate of change in elasticity, namely, higher *EV* reflects greater resistance to the (typical consumption-decreasing) effect of increasing unit prices.

As these study participants were in substance use disorder treatment, it is unsurprising that some individuals indicated no demand for opioids (*n* = 15 of 58) or alprazolam (*n* = 7 of 23) by providing all-zero consumption values across unit prices (i.e., non-participation). For these curves, *Q*_0_ and *EV* were recoded as 0; in these cases, the α parameter was treated as undefined/missing because it was infinitely high, reflecting low demand (104), and we used the *EV* parameter instead of α to retain a larger sample size for analysis.

For both opioid and alprazolam purchasing tasks, the binary variable “participation” (i.e., making non-zero vs. all-zero responses) and continuous parameters *r*^2^, *Q*_0_, α, and *EV* from demand modeling for each participant were exported into SPSS v27 to examine subgroup differences. Zero-bounded exponential modeling in GraphPad Prism was also used to generate subgroup-average demand curves for plotting (see figure captions for results of group-average curve fits).

For Aim 1, ANOVAs and chi-square tests were used to examine BZD misuse history group (never, >1 year ago, and past-year) differences in demographic, opioid use disorder treatment type, psychiatric diagnoses/medications, urinalysis results, medications, experimental opioid demand, affective dysregulation, and sleep-related measures. For Aim 2, ANOVAs, correlations, and multiple linear regression were used to examine associations of affective dysregulation and other measures with experimental BZD demand metrics.

## 3. Results

**FIGURE 1:**
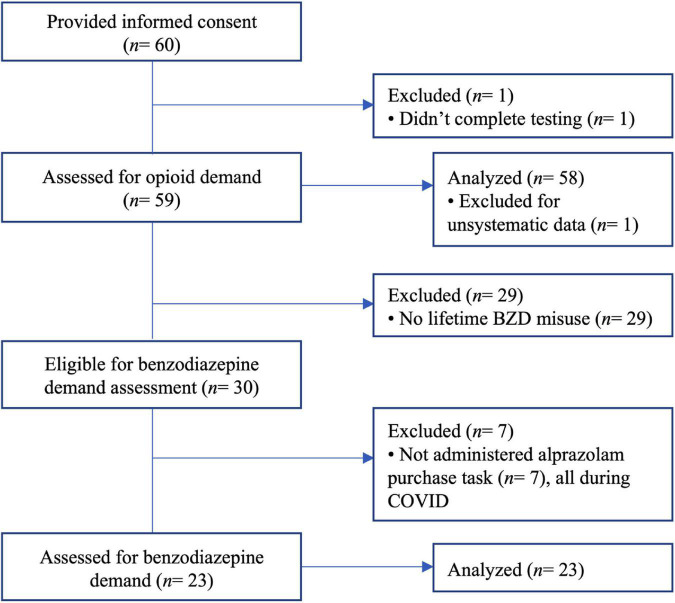
CONSORT diagram illustrating the flow of participants through the experimental procedures.

Figure 1 presents the CONSORT diagram for participant flow through the experimental procedures.

### 3.1. Aim 1: Differences between BZD misusers and never-misusers

Table 1 presents characteristics for the overall sample (*n* = 59) and by subgroups of participants who denied lifetime BZD misuse (*n* = 22), who misused BZDs > 1 year ago (*n* = 17) and who misused BZDs within the past year (*n* = 20), based on self-report from the *Drug History and Use Questionnaire* and *SCID* interview-based diagnosis of sedative disorder (see Section “2.2. Participant selection” for details). The subgroups significantly (*p* < 0.05) differed on several measures. Relative to never-misusers, lifetime BZD misusers (past-year and >1 year groups did not differ) were younger, more likely to be diagnosed with current major depressive disorder (with trends toward more depression symptoms on the BDI-II and likelihood of taking an antidepressant medication), and to present a urine sample that was opioid-negative and methadone-negative (with a trend toward more cocaine-negative samples).

**TABLE 1:** Participant characteristics [mean (SD) or percent (n)], stratified by BZD misuse group.

Measure	Total sample (*N* = 59)	Never misused BZD (*n* = 22)	BZD misuse > 1 year ago (*n* = 17)	BZD past-year misuser (*n* = 20)	Group χ^2^ or *F* (*p*)
***Demographics***					
Sex (M, F)	38, 21	14, 8	10, 7	14, 6	0.51 (0.775)
Race (B, W, other, missing)	31, 21, 3, 4	15, 4, 2, 1	7, 8, 0, 2	9, 9, 1, 1	7.08 (0.314)
**Age**	43.83 (13.27)	**52.25** (12.25)	**33.53** (7.65)	**43.00** (13.27)	**13.14 (<0.001)**
Education	12.03 (2.03)	12.36 (2.28)	11.35 (1.58)	12.25 (2.05)	1.37 (0.262)
Estimated IQ	105.48 (9.71)	107.57 (8.21)	101.65 (8.02)	106.45 (11.83)	2.00 (0.145)
***Treatment facility***					7.40 (0.494)
Acute residential	2% (1)	0% (0)	0% (0)	5% (1)	
Longer-term residential	14% (8)	5% (1)	19% (3)	21% (4)	
Transitional care	5% (3)	5% (1)	6% (1)	5% (1)	
Day program	47% (27)	45% (10)	44% (7)	53% (10)	
Other outpatient	32% (18)	45% (10)	31% (5)	16% (3)	
***Diagnoses [current (past-year)]***					
**Sedative use disorder**	19% (11)	**0%** (0)	**0%** (0)	**55%** (11)	**25.22 (<0.001)**
Alcohol use disorder	19% (11)	14% (3)	31% (5)	15% (3)	2.04 (0.360)
Stimulant use disorder	48% (27)	43% (9)	47% (7)	55% (11)	0.63 (0.732)
Cannabis use disorder	30% (17)	19% (4)	44% (7)	30% (6)	2.65 (0.266)
Anxiety disorder	21% (12)	10% (2)	25% (4)	30% (6)	2.55 (0.280)
Post-traumatic stress disorder	27% (15)	25% (5)	27% (4)	30% (6)	0.13 (0.937)
**Major depressive disorder**	26% (14)	**5%** (1)	**33%** (5)	**40%** (8)	**7.13 (0.028)**
Bipolar disorder	13% (7)	5% (1)	13% (2)	20% (4)	2.06 (0.358)
***Urinalysis results*** (+)					
BZD	15% (9)	9% (2)	12% (2)	25% (5)	2.28 (0.320)
Cocaine	20% (12)	36% (8)	12% (2)	10% (2)	5.58 (0.061)
**Opioids**	29% (17)	**50%** (11)	**18%** (3)	**15%** (3)	**7.71 (0.021)**
**Methadone**	53% (31)	**82%** (18)	**47%** (8)	**25%** (5)	**13.85 (<0.001)**
THC	10% (6)	5% (1)	12% (2)	15% (3)	1.32 (0.517)
***Medications (non-BZD)***					
MOUD agonist	71% (42)	86% (19)	71% (12)	55% (11)	5.03 (0.081)
Antidepressant	27% (16)	9% (2)	35% (6)	40% (8)	5.87 (0.053)
Analgesic	13% (7)	0% (0)	19% (3)	21% (4)	4.41 (0.110)
***Preferred-opioid demand***					
Participation (non-zero values)	75% (44)	77% (17)	77% (13)	70% (14)	0.34 (0.845)
Curve fit (*r*^2^)	0.93 (0.10)	0.89 (0.14)	0.95 (0.06)	0.94 (0.05)	1.74 (0.186)
*Q*_0_, non-normalized	19.33 (36.72)	14.31 (27.21)	26.85 (45.28)	18.22 (38.20)	0.55 (0.578)
*a*, non-normalized	0.1527 (0.8644)	0.3812 (1.4465)	0.0086 (0.0126)	0.0418 (0.0917)	0.81 (0.451)
Essential value, non-normalized	5.53 (12.70)	6.09 (18.66)	6.33 (9.17)	4.25 (6.64)	0.15 (0.861)
*a*, normalized	0.1423 (0.7607)	0.3170 (1.2621)	0.0354 (0.0529)	0.0498 (0.0635)	0.87 (0.427)
Essential value, normalized	3.80 (6.84)	3.94 (9.14)	4.09 (5.50)	3.38 (5.15)	0.06 (0.946)
***Affective dysregulation***					
SHAPS (anhedonia)	1.46 (2.15)	0.73 (1.35)	2.12 (2.96)	1.70 (1.92)	2.29 (0.111)
BDI-II (depression)	17.57 (11.44)	13.32 (10.60)	20.88 (11.60)	19.53 (11.16)	2.68 (0.078)
STAI Y1 (state anxiety)	48.12 (6.69)	47.48 (10.40)	48.29 (3.08)	48.65 (3.41)	0.16 (0.852)
**STAI Y2 (trait anxiety)**	43.77 (13.02)	**36.50** (10.70)	**48.35** (10.01)	**47.15** (14.63)	**5.65 (0.006)**
PSS (perceived stress)	30.03 (4.69)	28.81 (4.47)	31.12 (3.37)	30.40 (5.71)	1.24 (0.297)
ADUSE temptation	53.48 (18.79)	46.57 (20.12)	56.12 (15.72)	58.50 (18.44)	2.41 (0.099)
ADUSE confident	59.28 (20.63)	56.29 (24.22)	65.88 (15.88)	56.80 (19.77)	1.25 (0.295)
**DERS (emotion dysregulation)**	76.68 (28.66)	**61.01** (26.15)	**87.38** (26.33)	**85.35** (26.47)	**6.31 (0.003)**
DTS (distress tolerance)	3.28 (1.04)	3.60 (1.21)	3.24 (0.93)	2.96 (0.83)	2.06 (0.137)
**PASAT accuracy (# correct)**	88.19 (61.58)	**58.27** (51.49)	**105.53** (51.05)	**106.35** (69.26)	**4.66 (0.013)**
**PASAT quit %**	20% (12)	**36%** (8)	**6%** (1)	**15%** (3)	**6.03 (0.049)**
**Stroop positive latency (ms)**	769 (636)	**959** (824)	**558** (136)	**584** (266)	**3.21 (0.049)**
**Stroop negative latency (ms)**	811 (767)	**1,034** (934)	**608** (214)	**600** (281)	**3.43 (0.040)**
***Sleep/Behavioral alertness***					
*Epworth Sleepiness Scale*	8.98 (4.37)	8.18 (4.95)	8.88 (4.26)	9.95 (3.76)	0.86 (0.429)
*Insomnia Severity Index*	13.19 (7.42)	12.45 (7.47)	13.35 (6.47)	13.85 (8.36)	0.19 (0.831)
***Psychomotor Vigilance Task***					
#attentional lapses	9.85 (11.28)	13.36 (12.57)	6.94 (7.70)	8.45 (11.83)	0.81 (0.448)
Mean lapse reaction time (ms)	1,884 (9,275)	666 (875)	927 (2,389)	4,037 (15,798)	1.84 (0.169)
#false starts	9.12 (12.72)	9.14 (14.48)	8.12 (11.05)	9.95 (12.56)	0.09 (0.912)

M, male; F, female; B, black; W, white; BZD, benzodiazepine; THC, Δ^9^-tetrahydrocannabinol; MOUD, medications for treating opioid use disorder; SHAPS, Snaith-Hamilton Pleasure Scale; BDI-II, Beck Depression Inventory-II; STAI, State Trait Anxiety Inventory (Y1 = trait, Y2 = state); PSS, Perceived Stress Scale; ADUSE, Alcohol and Drug Use Self-Efficacy Scale; DERS, Difficulty in Emotion Regulation; DTS, Distress Tolerance Scale; PASAT, Paced Auditory Serial Addition Test; Stroop, Emotional Stroop task. Sedative use disorder diagnosis (DSM-5) and self-report of BZD misuse were to create the groups in this table, so this represents a manipulation check. Only non-BZD medications are reported because reasons for prescription are not being collected for overlapping indications involving BZDs (e.g., anxiety vs. insomnia). Bolded values indicate a significant overall group difference.

Relative to never-misusers, lifetime BZD misusers reported significantly higher scores for trait anxiety (STAI Y2 scale) and emotion regulation problem (DERS). Unexpectedly, lifetime BZD misusers had more correct responses and were less likely to quit task performance under cognitive duress (PASAT), and had faster response latencies during positive and negative affective interference trials (Emotional Stroop task). However, covariance analyses (ANCOVA) with age–which differed between BZD-misuser and never-misuser groups (see Table 1), found that these group differences in task performance were no longer significant, i.e., older age more parsimoniously explained longer response latencies (Stroop) and less accurate performance and more task quitting (PASAT). There were no other BZD misuse group differences on other measures, and presenting a BZD + urine sample was not associated with these measures.

Opioid demand curve fits were very high: 54 of 58 participants had *r*^2^ values > 0.80. Table 1 indicates that, based on the primary SPSS analysis of parameters that were computed from each participant’s demand curve (i.e., units of analysis), opioid demand intensity and essential value did not significantly differ for lifetime BZD misusers (>1 year ago or past-year) vs. never-misusers.

**FIGURE 2:**
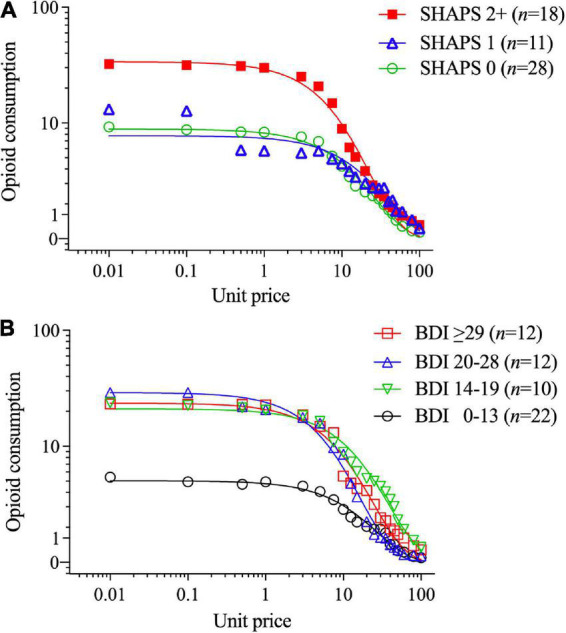
Opioid demand stratified by **(A)** Snaith-Hamilton Pleasure Scale (SHAPS) anhedonia total scores (0 vs. 1 vs. 2+) and **(B)** Beck Depression Inventory-II (BDI-II) scores (using clinical cutoff values). The primary SPSS analysis found significant SHAPS anhedonia group differences in opioid demand (see text, Section “3.1. Aim 1: Differences between BZD misusers and never-misusers”), and parameters computed in GraphPad Prism from group-average curves in Figure 1A confirm that the subgroup with SHAPS scores ≥ 2 compared to scores of 1 or 0 had higher opioid demand intensity (*Q*_0_ = 33.7 vs. 7.64 vs. 8.75, respectively), and essential value (*EV* = 7.75 vs. 2.98 vs. 2.36, respectively), *F*(1,18) = 84.2. The primary SPSS analysis did not find a significant BDI-II group difference in opioid demand intensity or essential value; however, parameters computed in GraphPad Prism from group-average curves in Figure 1B found that BDI-II scores indicating mild or greater depression severity (≥14) were associated with higher opioid demand intensity (*Q*_0_ = 4.9, 21.0, 29.0 and 23.5 for groups with scores of 0–13, 14–19, 20–28, and ≥ 29, respectively) and essential value (*EV* = 1.32, 8.24, 4.91, and 5.75, respectively), *F*(1,18) = 115.

In contrast, higher SHAPS anhedonia scores were significantly positively correlated with higher intensity of opioid demand (*Q*_0_, *r* = 0.59, *p* < 0.001), but not essential value (*r* = 0.19, *p* = 0.160). To refine the interpretation of these effects, participants were stratified into three groups based on SHAPS total scores (0, 1, or 2+), consistent with previous clinical studies and the observed distribution of scores in the present sample. Table 2 (upper section) and Figure 2A illustrate that those participants with SHAPS scores ≥ 2 had significantly higher opioid demand intensity, but not essential value, compared to subgroups with lower SHAPS scores. SHAPS scores and BDI-II scores were significantly correlated in the overall sample (*r* = 0.56, *p* < 0.001). Compared to SHAPS scores, BDI-II depression symptom scores showed a similar but weaker positive association with opioid demand intensity (*r* = 0.39, *p* = 0.002) and were not significantly associated with essential value (*r* = 0.20, *p* = 0.139).

**TABLE 2:** Parameter estimates for opioid and alprazolam demand, stratified by Snaith-Hamilton Pleasure Scale (SHAPS) anhedonia total scores.

Measure (Mean, SD)	SHAPS = 0	SHAPS = 1	SHAPS = 2+	Group χ^2^ or *F* (*p*)
Preferred-opioid demand	(*n* = 29)	(*n* = 11)	(*n* = 18)	
Participation (non-zero values)	**69%** (20)	**55%** (6)	**94%** (17)	**6.48 (0.039)**
Curve fit (*r*^2^)	0.92 (0.11)	0.93 (0.13)	0.93 (0.05)	0.28 (0.973)
Q_0_ (intensity)	**9.70** (19.29)	**7.18** (9.54)	**42.28** (55.17)	**6.02 (0.004)**
*a*, non-normalized	0.3055 (1.2846)	0.0087 (0.0097)	0.0328 (0.0847)	0.53 (0.592)
Essential value, non-normalized	4.19 (15.85)	3.62 (7.73)	8.86 (8.66)	0.90 (0.412)
*a*, normalized	0.2492 (1.073)	0.0618 (0.0627)	0.0192 (0.0477)	0.58 (0.566)
Essential value, normalized	2.75 (7.83)	3.04 (5.43)	5.93 (5.60)	1.30 (0.282)
Alprazolam demand	(*n* = 8)	(*n* = 7)	(*n* = 8)	
Participation (non-zero values)	75% (6)	43% (3)	88% (7)	3.69 (0.158)
Curve fit (*r*^2^)	0.93 (0.06)	0.98 (0.04)	0.90 (0.13)	1.35 (0.282)
*Q*_0_ (intensity)	5.82 (6.77)	2.30 (3.31)	36.81 (61.19)	2.11 (0.148)
*a*, non-normalized	0.0362 (0.3918)	0.0676 (0.0389)	0.0429 (0.1029)	0.18 (0.839)
Essential value, non-normalized	**0.77** (1.07)	**0.08** (0.12)	**4.62** (5.26)	**4.58 (0.023)**
*a*, normalized	0.0452 (0.0518)	0.0908 (0.0461)	0.0406 (0.0677)	1.77 (0.197)
Essential value, normalized	**0.90** (1.10)	**0.16** (0.12)	**3.72** (4.50)	**3.63 (0.045)**

Bolded values indicate a significant overall group difference.

Figure 2B illustrates that those participants with BDI-II depression symptom scores ≥ 14 (i.e., mild or greater depression severity) exhibited higher levels of opioid demand than those with lower BDI-II scores [≤13 indicates no clinical concern (87)]. SHAPS scores significantly correlated with several other measures of affective dysregulation (Table 3), however, these other measures were not related to opioid demand.

**TABLE 3:** Correlations between selected measures of affective dysregulation, insomnia severity and age in the overall sample (*N* = 59), and in parentheses, the subgroup of lifetime benzodiazepine misusers (*n* = 23).

	1	2	3	4	5	6	7	8	9	10
**SHAPS (anhedonia)**										
BDI-II (depression)	**0.56 (0.61)**									
STAI Y2 scale (state anxiety)	**0.57 (0.56)**	**0.86 (0.91)**								
PSS (perceived stress)	**0.31** (0.28)	**0.61 (0.69)**	**0.56 (0.71)**							
ADUSE temptation (to use drugs)	0.25 **(0.42)**	**0.41 (0.60)**	**0.62 (0.65)**	**0.42 (0.51)**						
DERS (emotion regulation problems)	**0.45 (0.56)**	**0.64 (0.74)**	**0.73 (0.84)**	**0.44 (0.62)**	**0.47 (0.68)**					
DTS (distress tolerance)	**−0.45 (−0.56)**	**−0.71 (−0.61)**	**−0.71 (−0.65)**	**−0.42 (−0.48)**	**−0.39 (−0.45)**	**−0.55 (−0.76)**				
ISI (insomnia severity)	**0.43 (0.59)**	**0.54 (0.53)**	**0.47 (0.50)**	**0.44 (0.43)**	**0.27** (0.29)	**0.50 (0.57)**	**−0.43 (−0.53)**			
PASAT accuracy (# items correct)	0.17 (0.24)	0.21 (0.23)	0.20 (0.29)	0.12 (0.05)	0.21 (0.31)	0.17 **(0.39)**	0.12 (−0.24)	−0.01 (0.25)		
PASAT quit (yes = 1)	**−0.29** (−0.14)	**−0.23** (−0.20)	−0.19 (−0.13)	−0.11 (0.04)	−0.10 (−0.23)	−0.12 (−0.01)	0.11 (0.04)	−0.08 (−0.09)	**−0.31** (−0.16)	
Age (years)	**−0.31** (−0.31)	**−0.42 (−0.64)**	**−0.45 (−0.62)**	**−0.51 (−0.50)**	**−0.49 (−0.48)**	**−0.43 (−0.58)**	0.15 (0.30)	**−0.29** (−0.37)	**−0.46 (−0.44)**	**0.40** (0.27)

Correlations in bold font are significant (*p* < 0.05). All correlations are Pearson *r* except PASAT quit (Kendall *tau*).

A multiple stepwise linear regression model with these two predictors found that only SHAPS anhedonia scores significantly predicted opioid demand intensity (standardized *beta* = 0.593, *t* = 5.46, *p* < 0.001) and explained 34.0% of the variance (adjusted *r*^2^), *F*(1,55) = 29.79, *p* < 0.001. SHAPS scores significantly correlated with younger age (*r* = −0.31, *p* = 0.018) and lower scores on the DTS (*r* = −0.42, *p* = 0.001), and with higher scores on STAI Trait Anxiety (*r* = 0.56, *p* < 0.001), and DERS (*r* = 0.45, *p* < 0.001), ISI (*r* = 0.45, *p* < 0.001), and PSS (*r* = 0.33, *p* = 0.008). Importantly, SHAPS scores singularly and significantly predicted opioid demand intensity when controlling for all these covariates, although adjusted *r*^2^ decreased to 23.3%, standardized *beta* = 0.497, *t* = 4.13, *p* < 0.001, *F*(1,52) = 17.06, *p* < 0.001.

In exploratory analyses, opioid demand metrics did not significantly differ when comparing males (*n* = 36) vs. females (*n* = 21), opioid injection users (*n* = 23) vs. non-injection users (*n* = 34), participants on opioid agonist therapy (methadone or buprenorphine, *n* = 41) vs. no agonist therapy (naltrexone or no medication, *n* = 17), participants in outpatient treatment (*n* = 44) vs. residential treatment (*n* = 9), nor participants with positive vs. negative urinalysis results, or presence/absence of substance use disorder and mental health diagnoses.

### 3.2. Aim 2: Differences within lifetime BZD-misusing subgroups

Among lifetime BZD misusers, alprazolam demand curve fits were very high: 22 of 23 participants had *r*^2^ values > 0.80. SHAPS anhedonia and BDI-II depression symptom scores, which were correlated in the overall sample, remained significantly correlated in this subgroup (*r* = 0.61, *p* < 0.001). In bivariate analyses, alprazolam demand intensity (*Q*_0_) significantly correlated with SHAPS scores (*r* = 0.69, *p* < 0.001) and marginally with BDI-II scores (*r* = 0.40, *p* = 0.058); and alprazolam essential value (*EV*) significantly correlated with both SHAPS scores (*r* = 0.46, *p* = 0.027) and BDI-II scores (*r* = 0.44, *p* = 0.034). SHAPS scores significantly correlated with several other measures of affective dysregulation in the subgroup of past-year BZD users (Table 3), however, these other measures were not related to alprazolam demand intensity or essential value. Although participant age was related to measures of affective dysregulation, age was not significantly related to BZD demand intensity or essential value. As we did for opioid demand, the same clinical cut-points were used to form SHAPS and BDI-II subgroups.

**FIGURE 3:**
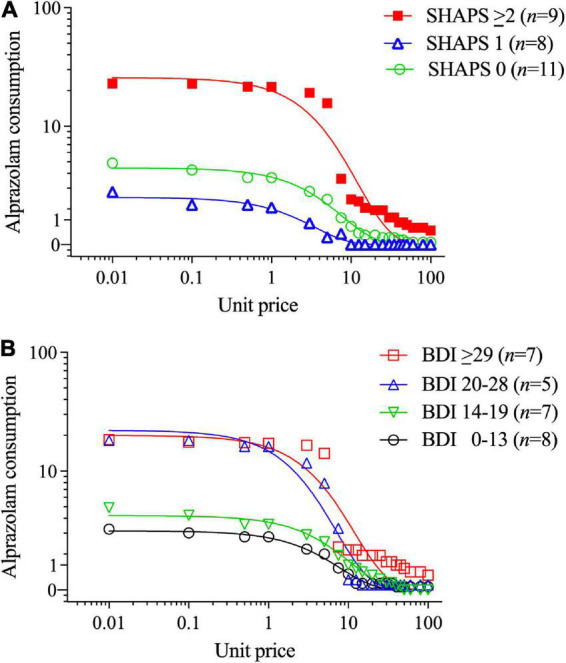
Alprazolam demand stratified by **(A)** Snaith-Hamilton Pleasure Scale (SHAPS) anhedonia total scores (0 vs. 1 vs. 2+), and **(B)** Beck Depression Inventory-II (BDI-II) depression total scores (using clinical cutoff values). The primary SPSS analysis found that participants with higher SHAPS anhedonia scores (≥2) and higher BDI-II depression symptom scores had significantly higher alprazolam demand intensity (see Section “3.2. Aim 2: Differences within lifetime BZD-misusing subgroups”). Parameters computed in GraphPad Prism from group-average curves in Figure 2A confirm that the subgroup with SHAPS scores ≥ 2 compared to scores of 1 or 0 had higher alprazolam demand intensity (*Q*_0_ = 25.7 vs. 2.11 vs. 4.22, respectively), and essential value (*EV* = 3.11 vs. 0.074 vs. 0.34, respectively), *F*(1,18) = 78.9. The primary SPSS analysis did not find a significant BDI-II group difference in alprazolam demand intensity or essential value; however, parameters computed in GraphPad Prism from group-average curves in Figure 2B found that progressively increasing BDI-II scores (0–13, 14–19, 20–28, and ≥ 29) were associated with monotonically increasing alprazolam demand intensity (*Q*_0_ = 1.92, 5.98, 15.7 and 19.9, respectively) and essential value (*EV* = 0.26, 0.37, 1.26, and 2.40, respectively), *F*(1,18) = 74.7.

Table 2 (lower section) and Figure 3 illustrate that those participants with SHAPS scores ≥ 2 (Figure 3A) and BDI-II scores ≥ 20 (indicating moderate to severe depression levels; Figure 3B) exhibited differences in alprazolam demand. A multiple stepwise linear regression model with these two predictors found that only SHAPS scores significantly predicted alprazolam demand intensity (standardized *beta* = 0.691, *t* = 4.38, *p* < 0.001) and explained 45.2% of the variance (adjusted *r*^2^), *F*(1,21) = 19.14, *p* < 0.001. A multiple stepwise linear regression model with these two predictors found that only SHAPS scores significantly predicted alprazolam essential value (standardized *beta* = 0.462, *t* = 2.39, *p* < 0.027) and explained 17.6% of the variance (adjusted *r*^2^), *F*(1,21) = 5.70, *p* < 0.027.

Alprazolam demand metrics did not significantly differ when comparing males (*n* = 14) vs. females (*n* = 9), injection opioid users (*n* = 11) vs. non-injection users (*n* = 12), participants on opioid agonist therapy (*n* = 13) vs. no agonist therapy (*n* = 10), those in outpatient treatment (*n* = 14) vs. residential treatment (*n* = 8), nor participants with positive vs. negative urinalysis results. Notably, presenting a BZD + urine sample (*n* = 7), reflecting recent use, was not significantly related to alprazolam demand. Although presence/absence of substance use disorder diagnoses and some mental health diagnoses was unrelated to alprazolam demand, there were two exceptions. First, presence (*n* = 8) vs. absence (*n* = 14) of major depressive disorder diagnosis was associated with greater alprazolam essential value (mean *EV* = 4.04 vs. 0.80), *F*(1,20) = 4.50, *p* = 0.047, with a trend toward higher demand intensity (mean *Q*_0_ = 36.5 vs. 4.5), *F*(1,20) = 3.90, *p* = 0.062, as well as higher symptom scores on SHAPS anhedonia (M = 3.75 vs. 0.57), *F*(1,20) = 18.70, *p* < 0.001, and BDI-II depression (M = 29.3 vs. 14.1), *F*(1,20) = 12.96, *p* = 0.002. Second, presence (*n* = 7) vs. absence (*n* = 15) of PTSD diagnosis was associated with greater alprazolam demand intensity (mean *Q*_0_ = 41.8 vs. 4.2), *F*(1,20) = 5.35, *p* = 0.032, and higher symptom scores on SHAPS anhedonia (M = 3.43 vs. 0.93), *F*(1,20) = 7.56, *p* = 0.012, and BDI-II depression (M = 31.3 vs. 14.2), *F*(1,20) = 17.84, *p* < 0.001.

**FIGURE 4:**
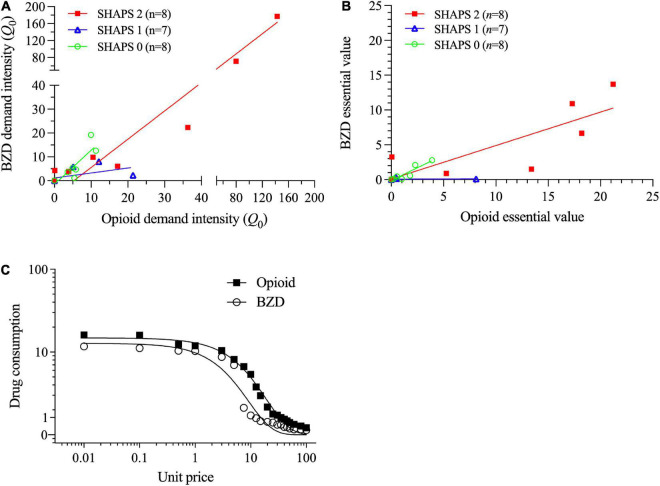
Within the subgroup of lifetime benzodiazepine (BZD) users, correlations of **(A)** opioid and benzodiazepine demand intensities (split axes enable better data visualization at low values) and **(B)** opioid and benzodiazepine essential values. Each of these two panels shows values separately for subgroups with 0, 1 or ≥ 2 Snaith-Hamilton Pleasure Scale (SHAPS) anhedonia scores. **(C)** In the primary SPSS analysis, opioid essential value (but not demand intensity) was significantly greater for the preferred-opioid than alprazolam (see Section “3.2. Aim 2: Differences within lifetime BZD-misusing subgroups”). In the secondary GraphPad Prism analysis, the preferred-opioid and alprazolam curves significantly differed (*Q*_0_ = 18.7 vs. 11.6; *EV* = 4.83 vs. 1.03), *F*(1,18) = 68.0, *p* < 0.01.

Benzodiazepine and opioid demand intensities were highly positively correlated (*r* = 0.98, *p* < 0.001; Figure 4A), as were BZD and opioid essential values (*r* = 0.86, *p* < 0.001; Figure 4B) and choice participation (χ^2^ = 27.00, *p* < 0.001). Regression slopes within each panel of Figure 4 indicate that opioid demand metrics were proportionally greater for the preferred-opioid than alprazolam. Repeated measures ANOVAs found that demand intensity was non-significantly higher for the preferred-opioid than alprazolam (*Q*_0_ = 16.1 vs. 15.5), *F*(1,22) = 3.98, *p* = 0.085; whereas, essential value was significantly higher for the preferred-opioid than alprazolam (EV = 4.10 vs. 1.90), *F*(1,22) = 6.83, *p* = 0.016. Figure 4C illustrates average demand curves (in the BZD-misusing group) for both the preferred-opioid and alprazolam.

## 4. Discussion

This ongoing study of persons being treated for opioid use disorder, and with polysubstance misuse histories, is examining factors that modulate economic demand for a standard BZD that is frequently misused (alprazolam) and each participant’s preferred misused opioid. The primary novel finding from this analysis is that participants who report multiple symptoms of anhedonia–a deficit in the experience and anticipation of pleasure–manifest significantly increased economic demand for both alprazolam and opioid drugs.

The first aim of the study was to determine whether BZD misuse history is related to affective dysregulation, opioid economic demand, and other clinically relevant measures. Based on systematic self-report and psychiatric diagnosis of sedative use disorder (involving a BZD), more than half of the sample (37 of 59 participants) were classified as having misused BZDs during their lifetime and over half of those (20 of 37) misused BZDs during the past year, whereas the remaining participants denied lifetime BZD misuse (22 of 59; comparison group). Relative to never-misusers, BZD misusers (both lifetime and past-year subgroups) were: higher on trait anxiety and emotion regulation problems and more likely to meet criteria for current major depressive disorder (consistent with our hypothesis); more likely to present opioid-negative and methadone-negative urine samples; and younger in age. In general, lifetime and past-year BZD misusers did not significantly differ on any of these measures; the only observed differences were between BZD misusing subgroups and the never-misuser group.

Surprisingly, BZD misusers did not significantly differ from never-misusers on several symptom measures of affective dysregulation including anhedonia (SHAPS), depression (BDI-II), state anxiety (STAI), distress tolerance (DTS), perceived stress (PSS), nor self-efficacy to resist substance use (ADUSE). Also, BZD misusers and never-misusers did not differ on current anxiety disorder, PTSD or bipolar disorder diagnoses (although the latter was infrequent) that are commonly linked to problems of affective dysregulation, nor did the groups differ on current non-sedative substance use disorder diagnoses.

Interestingly, BZD misusers and never-misuser groups did not significantly differ in experimental opioid demand. To our knowledge, this is the first clinical study to examine opioid demand in relation to differences in BZD-misuse history. By comparison, Petry and Bickel (66) examined simulated demand for heroin or the BZD diazepam in persons with a heroin-use history; most reported histories of injection use and all reported polysubstance use. Although all participants were in outpatient treatment and most were maintained on buprenorphine, all were instructed to imagine drug purchases while *not* receiving medication treatment (whereas such an instruction was not given in the present study). Those authors found that demand for heroin modestly decreased (i.e., was inelastic or relatively insensitive) in response to increases in its experimental price. In a separate assessment, it was found that as heroin price increased, diazepam purchasing increased. Thus, diazepam functioned as an economic substitute for heroin; yet, heroin purchases were found to be independent of diazepam prices, indicating asymmetrical substitution. In a recent study of persons in outpatient opioid agonist treatment for opioid use disorder, Schwartz et al. (68) found at a baseline visit that intensity of demand for BZD pills, which was lower than for heroin or cocaine in their sample, predicted the proportion of BZD-positive urine samples over a 6-month follow-up interval; thus, demand metrics were found to have clinical predictive value, which has also been demonstrated for treatment of tobacco use disorder (105), alcohol use disorder (106), and cocaine use disorder (107). Notably, Schwartz et al. (68) observed that opioid demand intensity (but not essential value) was significantly greater than for BZD pills, whereas the present study found that essential value for opioid vs. alprazolam significantly differed, with a trend for demand intensity. It is possible that (a) differences in the participant samples from the two studies, and/or (b) assessment of demand using the participant’s preferred opioid and route in the present study, played a role in these slightly discrepant findings.

The second aim of the present study was to investigate whether measures of affective dysregulation would modulate BZD demand within the subgroup of past-year BZD users. Anhedonia, which was associated with significantly greater opioid demand, was also found to significantly increase BZD demand. Therefore, in this sample, elevated anhedonia was a common predictor of increased drug demand. Interestingly, current depression symptom levels (BDI-II) showed similar, but slightly weaker, effects than anhedonia on both opioid and BZD demand. This raises an important question of behavioral specificity. Anhedonia symptoms measured with the SHAPS represents a more narrow phenotype (positive hedonic deficit) than depression symptoms measured with the BDI-II. Although the Beck Depression Inventory has three items (#4, loss of pleasure; #12, loss of interest; #21, loss of interest in sex) that have been proposed to measure anhedonia (75) the majority of items focus on negative-affective symptoms and neurovegetative signs of affective disorder. Thus, anhedonia more precisely captures impairment of positive reinforcement. Interestingly, anhedonia but not depression was found to predict cocaine use in a clinical trial (108), in support of its distinct construct and predictive validity.

Anhedonia has been associated with impaired reinforcement learning (82, 109, 110). Thus, for persons with higher (vs. lower) anhedonia, repeated drug use and conditioning may strengthen drug demand to a greater degree so that it becomes more intense (at low prices, *Q*_0_) and resistant to price increases (inelastic, or higher essential value). This phenotype maps onto some proposed sub-domains of anhedonia, e.g., approach motivation, reward valuation, effort valuation/willingness to work, and habit formation (111, 112). Although anhedonia might generally increase drug demand (as we found for opioid and alprazolam), it is conceivable that anhedonia might also interact differently across misused drugs [but see (113) for interpretive complexities]. Notably, laboratory animal models of drug self-administration have found that GABAergic agents including BZDs and alcohol can produce anti-conflict effects, i.e., they disinhibit punished behaviors (114–117) and this could enhance the expression of risky behaviors (118). For people who have experienced adverse consequences of opioid and sedative polydrug use (119, 120), i.e., such use has been punished or suppressed, BZDs (and alcohol) may interfere with efforts to abstain. Further research might explore whether this BZD anti-conflict effect could be enhanced in persons with higher anhedonia and may also interact with the behavioral cost of the drug.

The neural substrate for anhedonia is hypothesized to involve disruptions to a cortical/subcortical neural circuit whereby elevated prefrontal cortical excitability leads to decreased striatal dopamine activation (79, 121–123). Interestingly, opioid withdrawal-related anhedonia in rats (increased intracranial electrical self-stimulation) was associated with reduced vulnerability to subsequent morphine self-administration (124). However, in samples of patients with opioid use disorder, anhedonia has been variously found to correlate with recent opioid use during medication treatment but not during long-term abstinence (125, 126) as well as drug-cue or natural reward cue-reactivity during opioid abstinence (127–129) but not in all studies (130); these mixed findings imply that elevated anhedonia may be a dissociable phenotype from opioid or other substance use/abstinence [cf. (131)].

In a preclinical study, rats withdrawn from BZD exposure exhibited reduced preferences for both a cage compartment that had been paired with a sexual odor cue and for a context previously paired with amphetamine–a pattern of attenuated reward-seeking behaviors suggestive of increased anhedonia (132). In humans, there are very few studies of BZDs and anhedonic symptoms. Use of BZDs among patients with major depressive disorder was found to be associated with increased anhedonia but not anxiety or depression symptom levels (perhaps because BZDs mitigated anxiety) and anhedonia was the strongest predictor of BZD use in that study (133). A recent clinical study of repeated ketamine infusions in 42 patients with treatment-resistant major depression found that ketamine (an NMDA receptor antagonist) significantly reduced anhedonia (SHAPS scores) after each infusion but only among the subgroup of patients who did not use BZDs (134). Although the present study was not designed to examine BZD withdrawal and anhedonia, we did not find any significant difference in anhedonia scores between BZD misusers and never-misusers, nor between participants whose urine samples tested BZD-positive vs. BZD-negative. Further research is needed to understand the relationship between BZD use/discontinuation and anhedonia.

Benzodiazepines modulate activity to varying degrees at GABA_*A*_ receptor subtypes which differentially correlate with their reinforcing, sedative/hypnotic and myorelaxant properties (60, 63, 135, 136). In persons with opioid use disorder, BZDs might also (e.g., *via* GABA interneurons on *mu*-opioid receptors) indirectly modulate *mu*-opioid receptor function (137), potentially leading to altered sensitivity to drug reinforcement (138). In the present study, neither past-year BZD misuse nor BZD-positive urine samples were related to opioid demand; however, only 15% of the overall sample had BZD-positive samples so there is likely insufficient statistical power to detect an effect. Unfortunately, we lack systematic data on the precise temporal pattern of BZD and opioid use (e.g., simultaneous vs. concurrent), which could potentially influence these results.

We also note that several measures were not significantly related to BZD misuse group, anhedonia or depression, nor opioid or BZD demand. These include some demographic factors (e.g., notably, no sex differences), psychiatric comorbidities other than sedative use disorder, and several measures of negative affective dysregulation. Absence of effects of insomnia and negative affective disturbance–which are often related (44, 139)–was surprising. However, we did find that younger age was significantly related to several measures of affective dysregulation (SHAPS, BDI-II, STAI, ADUSE, DTS, DERS, PASAT), both in the overall sample and within the past-year BZD-use group (see Table 3), but age was not significantly related to BZD demand metrics.

This study has several limitations. First, there is a relatively small sample size, although our sample is not smaller than others’ comparable work (66, 68). Notably, our planned enrollment is expected to be up 120 participants, so we will have ample power to examine these and other effects in greater detail. Second, we conducted sensitivity analyses that excluded individuals with zero participation in the purchase task (leading to reduction in group size); these analyses suggest that alprazolam demand metrics should be cautiously interpreted, whereas censoring of participants with zero participation did not significantly alter opioid demand metrics. Third, we are recruiting individuals from various treatment settings/modalities to increase the heterogeneity and population representativeness of the sample with regard to polysubstance use and types of interventions; although this introduces variance that may complicate interpretation of the findings, we believe it can improve the generalizability of findings to treatment settings and prompt new hypotheses for investigation. Fourth, it is presently not feasible to collect reliable data on medication treatment doses, which could affect opioid demand and perhaps BZD demand. Notably, it has been shown in laboratory animal models that acute pretreatment with morphine, buprenorphine or naltrexone can increase fentanyl demand elasticity, i.e., decrease essential value (140). Fifth, unlike Petry and Bickel (66), we did not examine cross-price elasticity between the preferred-opioid and alprazolam in this study; although we designed such a manipulation, this was ultimately excluded due to the length of the overall assessment battery (several additional measures in this battery are not reported here). Finally, consistent with the work by Schwartz et al. (68), we are interested in whether these demand measures can predict longer-term outcomes. In the present study, we are collecting 3-month follow-up measures; however, at this time, these data are too sparse for meaningful analysis. However, it should be noted that purchasing “participation” (i.e., making non-zero drug choices at any price) in an in-treatment population may indicate the presence of a relapse risk. Thus, in future research, it could be useful to include participation as well as demand intensity and essential value metrics when reporting results with samples of patients.

In conclusion, this study identifies increased anhedonia as a shared factor for greater economic demand of opioid and BZD drugs in persons with histories of polysubstance use who are being treated for opioid use disorder. Anhedonia, which has commonly received attention in psychiatric disorders especially major depressive disorder [e.g., (75, 77, 141)], has a biological basis partly independent of depressive symptoms (142–144). Anhedonia has been observed during acute and protracted drug abstinence and may be related to drug craving (78). Thus, anhedonia may play an important predictive role in substance use disorder treatment outcome. Based on our findings that anhedonia can modulate drug demand, along with recent findings that experimental demand can predict treatment outcome in substance use disorders (68), we believe that it could be useful to routinely include assessments of anhedonia and hypothetical drug demand in clinical settings to monitor the progress and recovery of persons with these disorders.

Future directions are to understand the multidimensional nature of affective dysregulation in this population, develop improved biomarkers/phenotypes to predict clinical outcomes and, from this improved understanding, develop behavioral, medication and neuromodulation interventions to reduce anhedonia and improve treatment efficacy (111, 141, 145–147).

## Data availability statement

The original contributions presented in this study are included in the article/supplementary material, further inquiries can be directed to the corresponding author.

## Ethics statement

The studies involving human participants were reviewed and approved by Human Investigation Committee, Wayne State University. The patients/participants provided their written informed consent to participate in this study.

## Author contributions

MG acquired funding, designed the study, oversees data collection, conducted the analyses, and drafted the manuscript. TM, LL, and TR contributed to study design and assessments and edited the manuscript. TM conducted psychiatric interviews and collected data. LL supervised psychiatric assessments. All authors taken responsibility for the content of the manuscript.

## Footnotes

1
https://ibrinc.org/behavioral-economics-tools/
